# Integrating generative AI and physical tests to strengthen claims about emergent phenomena

**DOI:** 10.1073/pnas.2529610122

**Published:** 2025-11-17

**Authors:** Weihao Cheng, Zekai Yu

**Affiliations:** ^a^School of Computer Science and Technology, Hangzhou Dianzi University, Zhejiang Province 310018, People’s Republic of China; ^b^School of Communication Engineering, Hangzhou Dianzi University, Zhejiang Province 310018, People’s Republic of China

We read with great appreciation the Perspective by Tiwary et al. (1), which provides a timely and insightful roadmap for integrating generative AI into computational chemistry. The authors’ emphasis on connecting AI with statistical mechanics and thermodynamics represents an important step toward truly predictive molecular modeling. Having carefully studied the article, we would like to offer a few collegial and constructive suggestions to make the framework even more robust and reproducible.

The authors insightfully note that current generative models “excel at interpolation but struggle with emergent behaviors” (p. 2). To make this observation more tangible, we suggest including quantitative benchmarks for rare-event prediction. For instance, models could be evaluated on reproducing transitions with probabilities below 10^−5^or free-energy barriers exceeding 5 kcal·mol^−1^, values typical of catalytic or conformational processes (2). Reporting whether transition-state free energies are reproduced within ±1 kcal·mol^−1^ or committor probabilities within 10% of molecular dynamics (MD) references would turn this conceptual limitation into a measurable and comparable performance indicator (3).

The Perspective also highlights the need for interpretability and reliability testing. Including ensemble variance or Bayesian posterior width for energy predictions could quantify model confidence, while calibration plots of predicted versus empirical errors would reveal how well uncertainties are estimated. Quantitative divergence measures—such as the Wasserstein or KL distance—between generated and reference Boltzmann ensembles could further clarify how closely the generative distributions approximate physical equilibria (4). These diagnostics would transform interpretability into a reproducible evaluation practice rather than a qualitative notion.

Regarding data scarcity and generalization beyond the training distribution, the manuscript insightfully notes that “more data are not always better.” We encourage the authors to highlight how hierarchical or transfer-learning strategies might help. A short case study showing how fine-tuning on small ab initio datasets reduces energy Rms errors—from about 2 kcal·mol^−1^ to below 1 kcal·mol^−1^, for example—would concretely illustrate the benefits of incorporating high-fidelity data (5). Even approximate quantitative examples would make the discussion more instructive for researchers building data-efficient generative models.

Finally, the section on hybrid AI–physics frameworks such as AF2RAVE and thermodynamic maps could be made more tangible through small demonstration pipelines. One might involve a simple proton transfer in water (barrier ≈ 6 kcal·mol^−1^), and another a protein DFG-in/out conformational transition (ΔG ≈ 3 to 5 kcal·mol^−1^). For each, showing the raw generative output, the physics-based refinement or reweighting, and comparison with MD reference observables such as rmsd or population ratios would convincingly illustrate the practical realizability of the proposed approach (6–8).

Overall, Tiwary et al. have offered an ambitious and intellectually rigorous roadmap. By incorporating a few targeted benchmarks, uncertainty analyses, and demonstration pipelines, the Perspective could become a definitive reference for building physically grounded generative AI in chemistry.

## Compliance with Ethical Standards

### Research Involving Human Participants and/or Animals.

This research did not require the involvement of human or animal subjects. Therefore, ethical approval for this study was not required according to local regulations and institutional policies.

### Informed Consent.

Not applicable.
